# Hydrophobically Associating Polyacrylamide “Water-in-Water” Emulsion Prepared by Aqueous Dispersion Polymerization: Synthesis, Characterization and Rheological Behavior

**DOI:** 10.3390/molecules28062698

**Published:** 2023-03-16

**Authors:** Yongli Lv, Sheng Zhang, Yunshan Zhang, Hongyao Yin, Yujun Feng

**Affiliations:** 1 Petroleum Engineering Technology Research Institute of Shengli Oilfield, SINOPEC, Dongying 257000, China; 2 Polymer Research Institute, State Key Laboratory of Polymer Materials Engineering, Sichuan University, Chengdu 610065, China; 3 Shengli Oilfield Shengli Chemicals Co., Ltd., Dongying 257055, China

**Keywords:** hydrophobically associating polyacrylamide, “water-in-water” emulsion, aqueous dispersion polymerization, rheological behavior

## Abstract

The hydrophobically associating polyacrylamide (HAPAM) is an important kind of water-soluble polymer, which is widely used as a rheology modifier in many fields. However, HAPAM products prepared in a traditional method show disadvantages including poor water solubility and the need for hydrocarbon solvents and appropriate surfactants, which lead to environmental pollution and increased costs. To solve these problems, we reported a novel kind of HAPAM “water-in-water” (*w*/*w*) emulsion and its solution properties. In this work, a series of cationic hydrophobic monomers with different alkyl chain lengths were synthesized and characterized. Then, HAPAM *w*/*w* emulsions were prepared by the aqueous dispersion polymerization of acrylamide, 2-methylacryloylxyethyl trimethyl ammonium chloride and a hydrophobic monomer. All these emulsions can be stored more than 6 months, showing excellent stability. An optical microscopy observation showed that the particle morphology and the particle size of the HAPAM emulsion were more regular and bigger than the emulsion without the hydrophobic monomer. The solubility tests showed that such HAPAM *w*/*w* emulsions have excellent solubility, which took no more than 180 s to dilute and achieve a homogeneous and clear solution. The rheology measurements showed that the HAPAM association increases with a hydrophobe concentration or the length of hydrophobic alkyl chains, resulting in better shear and temperature resistances. The total reduced viscosity was 124.42 mPa·s for cw101, 69.81 mPa·s for cw6-1, 55.38 mPa·s for cw8-0.25, 48.95 mPa·s for cw12-0.25 and 28 mPa·s for cw16-0.25 when the temperature increased from 30 °C to 90 °C. The cw8-2.0 that contains a 2 mol% hydrophobe monomer has the lowest value at 19.12 mPa·s due to the best association. Based on the excellent stability, solubility and rheological properties, we believe that these HAPAM *w*/*w* emulsions could find widespread applications.

## 1. Introduction

The hydrophobically associating polyacrylamide (HAPAM) is an important kind of water-soluble polymer, which has been extensively studied over the past few decades [1,2,3,4,5,6]. A single HAPAM molecule contains a small number of hydrophobic groups (generally less than 2 mol%) in the main polyacrylamide chain [2]. Because hydrophobic groups tend to exclude water, HAPAM aqueous solutions exhibit intermolecular hydrophobic interactions above a certain concentration, i.e., the formation of transitional three-dimensional networks of polymer chains. They also exhibit rheological behavior, such as thermic thickening and high viscosity. The dynamic junctions are also disrupted upon high shear stress but reform when the force ceases [7,8,9,10,11,12,13,14,15]. The HAPAM has been used in many applications as a thickening agent, including fracturing, drilling, latex paint systems, pigment printing for textiles, coatings and cosmetics [4]. The preparation method plays a crucial role in the development of the HAPAM. Although many efforts have been devoted to the preparation of the HAPAM, challenges still remain.

Two important synthetic methods have been utilized to prepare the HAPAM: chemical post-modification and copolymerization [7,11]. Deguchi and Lindman [16] reported that the HAPAM can be prepared by the direct modification of a low-molecular-weight polyacrylamide (PAM) and an alkyl bromide in dimethyl sulfoxide (DMSO). This work was followed by Feng et al. [7] with the modification of a higher-molecular-weight PAM with octyl bromide in DMSO. One can achieve favorable characteristics by post-modification, such as the statistical arrangement of hydrophobes [17] and parameter control in the hydrophobic group content or hydrophobe structure while keeping the molecular weight and hydrophobic group distribution constant. Unfortunately, these products are often dry and are difficult to dissolve, which encumbers their use in applications. For example, it usually takes weeks to dissolve dry powders of the HAPAM in water [18]. Copolymerization is a frequently used method that includes inverse emulsion, microemulsion and micellar polymerization. Pabon [19] synthesized AM-sodium acrylate (NaAA) and an amphiphilic comonomer (or surfmer), isooctylphenoxy-poly(oxyethylene)(n) methacrylate copolymers, by inverse emulsion polymerization. They attained a 25 wt% solid content in their emulsions. Subsequently, they studied inverse emulsion polymerization routes to synthesize the AM and *N*,*N*-dihexylacrylamide or *N*,*N*-diphenylacrylamide copolymers [13]. However, the HAPAM emulsions prepared by inverse emulsion polymerization have insufficient stabilities for long-term storage [14]. To provide a solution to this problem, Lu and Feng [20] used inverse microemulsion polymerization to synthesize copolymers of AM and cationic hydrophobic monomers. Presently, the commonly accepted method is micellar copolymerization, in which the hydrophobic comonomer is dissolved, forming micelles by adding an appropriate surfactant [9]. Using this copolymerization technique, the copolymers of AM, monoalkylcarylamide or dialkylcarylamides with other monomers [9], the copolymers of AM and *N*,*N*-dihexylacrylamide [12], the copolymers of AM and *N*-4-ethylphenyl-acrylamide or *N*-4-ethylphenyl-propionamide [15] and the copolymers of AM and triecyl acrylate [14] were prepared. However, the final products produced by micellar copolymerization generally have high viscosity even with a low solid content, so it is difficult to produce high solid content products based on this method. The common disadvantages in post-modification and copolymerization include the poor water-solubility of the polymers and the need for hydrocarbon solvents and appropriate surfactants, which lead to environmental pollution and increased costs. Therefore, there is an ongoing need for the development of HAPAMs that are environmentally friendly, safe and have good water-solubility and viscosity properties for use as drilling fluids.

Aqueous dispersion permits the production of the polymers as stable colloids in water. During the homogeneous dispersion polymerization process, the water-soluble monomers were dissolved in an aqueous brine solution and were then polymerized by using a water-soluble free radical initiator. The high-molecular-weight polymer was deposited from the homogeneous solution due to the salt-out effect and then captured by a low-molecular-weight stabilizer and dispersed to form a dispersion system that could dissolve rapidly in water. The resulting dispersion system is called a “water-in-water” emulsion because its appearance looks like an emulsion. In the practical applications, the dispersion was either diluted with water without any surfactant inverter or simply added into a wastewater stream without costly equipment, thereby diluting the salt and allowing the polymer to dissolve and become a clear, homogeneous polymer solution.

In the early 1980s, Hosoda et al. [21] first reported dispersion polymerization in aqueous media. In their work, NaAA was polymerized in the presence of poly(ethylene glycol) to obtain a poly(sodium acrylate) “water-in-water” emulsion. Jaeger et al. [22] then reported the dispersion copolymerization of acrylamide and the cationic monomer, benzyl-2-(methacryloyloxy)ethyldimethyl ammonium chloride (MADAMBQ), in aqueous salt media with a block copolymeric stabilizer. They found that the emulsion was stable up to several months. They also investigated the dispersion homopolymerization of methacryloyloxyethyl trimethyl ammonium chloride (MADAM) and MADAMBQ [23]. In subsequent work, Reichert et al. utilized poly(ethylene oxide) as a stabilizer to homopolymerize MADAMBQ in a NaCl aqueous solution [24]. Cho et al. [25] carried out the dispersion polymerization of AM in aqueous ammonium sulfate using a low-molecular-weight polycation as a stabilizer, and stable emulsions were prepared. In their later work, aqueous dispersions of a cationic polymer were prepared by the dispersion copolymerization of AM and acryloyloxyethyl dimethylbenzyl ammonium chloride (AODBAC) in an aqueous solution of ammonium sulfate and in the presence of a cationic homopolymer stabilizer [25]. Recently, Wang et al. [26] reported the dispersion copolymerization of AM with 2-methylacryloylxyethyl trimethyl ammonium chloride (DMC) in an aqueous salt solution containing ammonium sulfate and sodium chloride with a polycationic stabilizer. Similar work was also reported by Wu et al. [27]. They copolymerized AM and DMC in an aqueous ammonium sulfate solution with a PDMC homopolymeric stabilizer. Although there have been some investigations for the synthesis of a PAM by aqueous dispersion polymerization techniques, the preparation of the HAPAM by this technique has not been documented so far. Due to the high stability and good dispersion of the products, the aqueous dispersion polymerization technique may solve the above long-term problems in the post-modification and copolymerization of the HAPAM.

Inspired by this fact, we utilized aqueous dispersion polymerization techniques to synthesize the hydrophobically associating polyacrylamide. The hydrophobic monomer 2-methylacryloylxyethyl dimethylalkylammonium bromine was synthesized and then copolymerized with AM and DMC by aqueous dispersion polymerization to afford a series of HAPAM “water-in-water” emulsions. The morphology of the particles, the solubility and the rheological properties were then investigated.

## 2. Results and Discussion

### 2.1. Synthesis and Characterization

The aqueous dispersion polymerization technique has attracted increased attention in recent years due to its advantages. Polymerization occurs in an aqueous solution containing a low-molecular-weight polymer stabilizer and organic salt. During the polymerization process, high-molecular-weight polymers are produced, which further form solid particles due to the salt-out effect and are deposited from the solution and then captured by a pre-added stabilizer. The solid content can reach around 30.0% and the stability can keep for several months. In comparison with other emulsion products, this product does not contain organic solvents so that it is less harmful. Moreover, the particles can be rapidly dissolved in water, showing a much better solubility than traditional powder products. The aqueous dispersion polymerization technique has been used to prepare the PAM *w*/*w* emulsion, but the HAPAM *w*/*w* emulsion had not been developed.

To obtain more information about hydrophobically associating PAM *w*/*w* emulsions and the incorporation behavior of diluted copolymer solutions, a series of copolymers containing various monomers were prepared and investigated. The recipes for the copolymers are shown in Table 1. It was found that the hydrophobic monomers played a crucial role in the synthesis of the *w*/*w* emulsions. Comonomers with shorter alkyl chains, such as 2-mntethylacryloylxyethyl dimethylbutylammonium bromine (DM-4), 2-methylacryloylxyethyl dimethylhexylammonium bromine (DM-6) or 2-methylacryloylxyethyl dimethyloctylammonium bromine (DM-8), can be used at higher concentrations (2 mol%). Due to stronger associative properties, the higher concentration of a comonomer with shorter alkyl chains (C_12_–C_16_) resulted in the instability of the emulsions. Therefore, the hydrophobic monomer content could not exceed 0.5 mol% in most experiments. All the emulsions shown in Table 1 had low viscosities and can be stored for more than 6 months. This excellent stability might be related to the surface properties of the emulsions, which will be investigated in our future work, i.e., to measure the surface wettability on various treated solids using inexpensive contact angle goniometers that perfectly work with either smartphones [28] or USB microscopy cameras [29].

**Figure 1:**
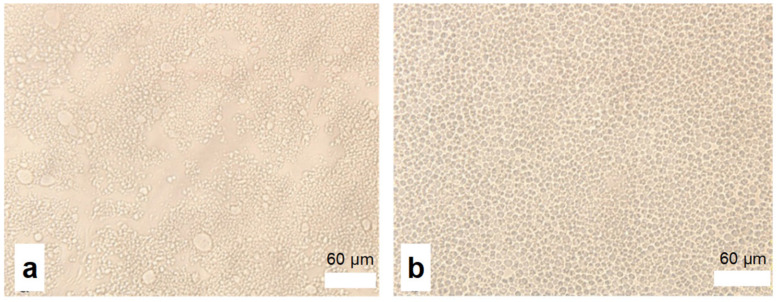
Micrographs of the copolymer particles in a “water-in-water” emulsion. (**a**) P (AM-*co*-DMC) (CW101); (**b**) P (AM-*co*-DMC-*co*-DM8) (CW8-0.5).

The particle morphologies of hydrophobically associated aqueous dispersions were characterized by an optical microscope and compared with the P(AM-*co*-DMC) dispersion. As evidence of dispersion, the morphology of the particles formed in dispersion polymerization was observed by different research groups. For example, Song et al. [25] and Wang et al. [26] observed the particle shapes of the dispersions. Figure 1 shows the micrographs of P(AM-*co*-DMC) (Figure 1a, CW101) and a typical hydrophobically associating polyacrylamide *w*/*w* emulsion (Figure 1b, CW8-0.5). It is found that the particle shape of the CW8-0.5 was more regular than that of CW101. The particle size of CW8-0.5 was about 5 μm, which is bigger than that of CW101. One reason may be the association of the copolymers: polymer chains in particles form tiny three-dimensional network configurations, so the particle’s volume is bigger.

**Figure 2:**
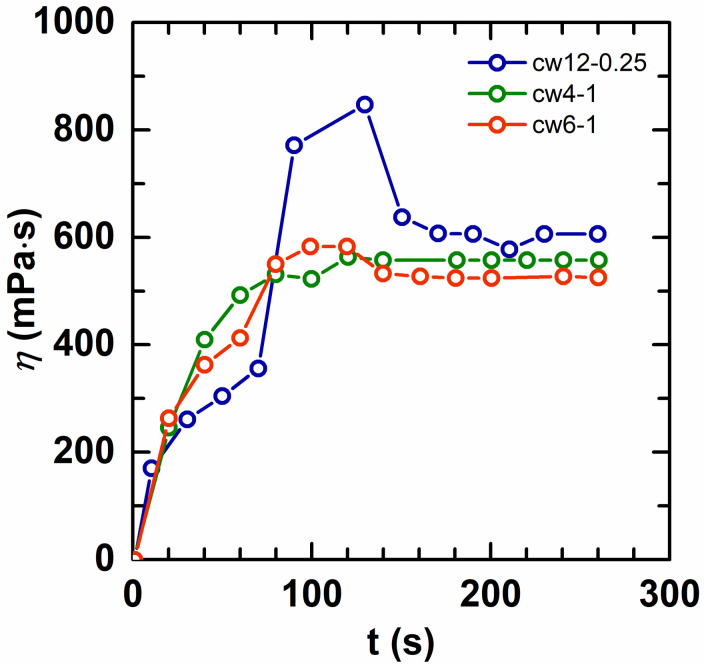
The solubility of HAPAM *w*/*w* emulsion in water. The polymer concentration is fixed at 0.5%.

An important advantage of aqueous dispersion polymerization is the good solubility in water. One of the main purposes of this work is to improve the solubility of the HAPAM in water using aqueous dispersion polymerization, so the solubility of HAPAM *w*/*w* emulsions was investigated. Figure 2 shows that all the HAPAM *w*/*w* emulsions have good solubility in water. For all the HAPAM *w*/*w* emulsions, they took no more than 180 s to obtain homogeneous and clear solutions after dilution, suggesting a rapid dissolution rate. Note that the viscosity experienced a dramatic increase at around 100 s, and then reduced to a stable value. This phenomenon became more obvious with the increase in the hydrophobic alkyl chain length. This can be explained as the hydrophobic alkyl chain forms a transient physical cross-linked network during the dissolving process. When the chains were completely hydrated with the time increase, their physical cross-linking reached an equilibrium state so that the viscosity achieved a stable value.

### 2.2. Compositional Analysis

In some articles, the hydrophobic group in the copolymer was determined by ^1^H NMR [7,9,10,11,12,13,20], UV [14,15], ^13^C NMR [20], FT-IR [20] spectroscopy, etc. However, in our work, the content of the hydrophobe was too low in the copolymers, and the molecular structure of the hydrophobe was similar to that of another monomer DMC. Therefore, it was difficult to determine the polymer composition using these methods. In this work, ion change chromatography (IC), thermogravimetry (TG) and differential thermal gravity (DTG) techniques were used for the qualitative analysis of the polymer composition.

#### 2.2.1. IC Analysis

**Figure 3:**
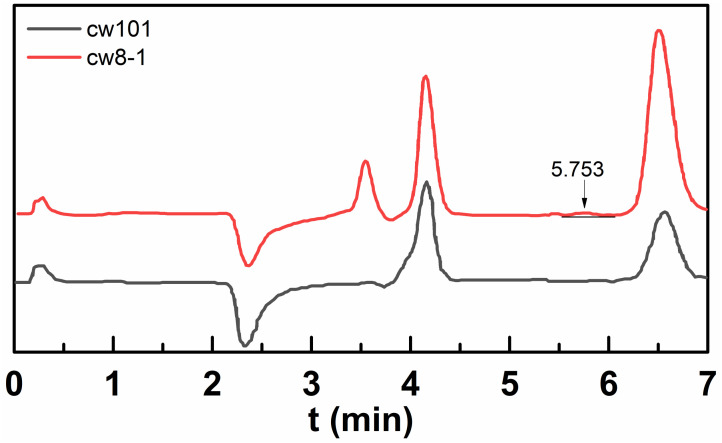
The IC spectra of cw101 and cw8-1 polymers emulsions.

Generally, the bromine element in the copolymers can be determined by IC. However, apart from bromine, there are a large number of chlorides in the polymer chain from DMC. Chloride and bromide have very similar characteristics in an IC analysis, so the content of Br^−^ cannot be accurately determined. Therefore, the result of the IC analysis only provided evidence that hydrophobes exist in the polymers. Figure 3 shows the IC spectrum of cw101 (without hydrophobes), where no signal of Br^−^ was found, which is consistent with the molecular structure. On the contrary, the characteristic peak of Br^−^ was observed at around 5.50 min in the IC spectrum of cw8-1, suggesting the presence of a hydrophobe. However, the Br^−^ signal was largely affected by the signals of Cl^−^ and NO_2_^−^, and its calculated amount was only 0.12 wt%, much lower than the theoretical value of 0.72 wt%. Though the signature of Br^−^ was interfered, the comparison result of the IC spectra of cw101 and cw8-1 confirms the presence of a hydrophobic group in cw8-1.

#### 2.2.2. TG and DTG Analysis

**Figure 4:**
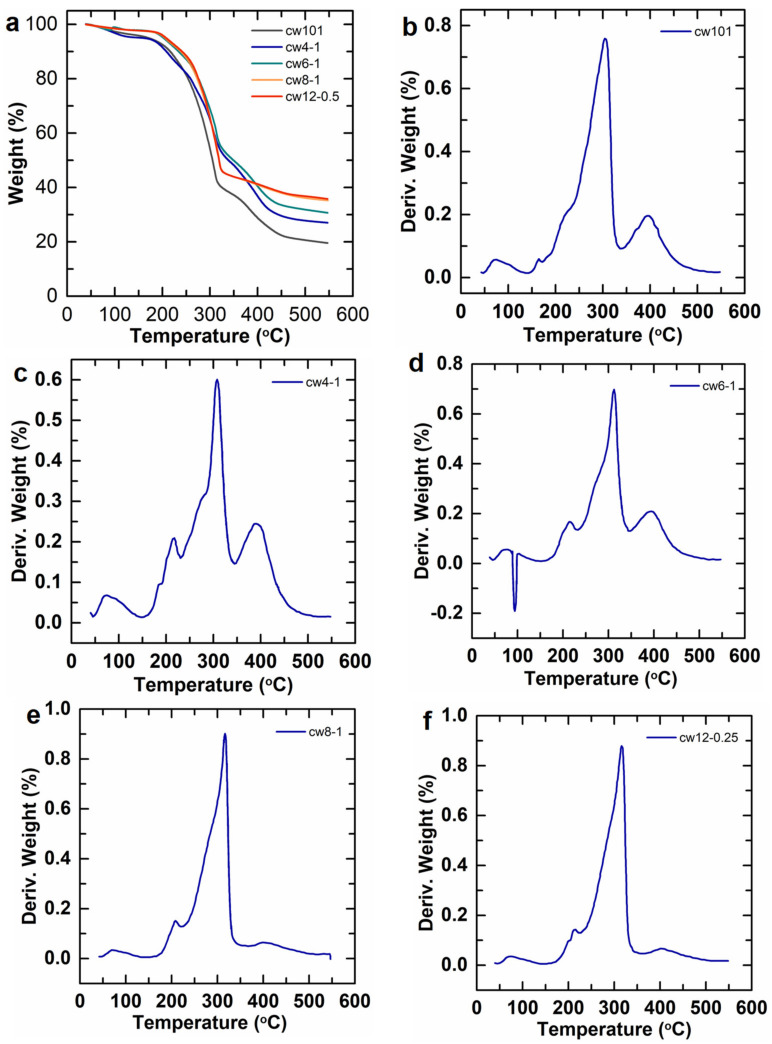
The TG and DTG thermal curves for HAPAM and P(AM-*co*-DMC). (**a**) TG thermal curves for polymers; (**b**) DTG thermal curves for cw101; (**c**) DTG thermal curves for cw4-1; (**d**) DTG thermal curves for cw6-1; (**e**) DTG thermal curves for cw8-1; (**f**) DTG thermal curves for cw12-0.25.

Although the hydrophobic content was very low, one can note the great difference in the TG and DTG spectra between the HAPAM and P(AM-*co*-DMC) samples. The TG data of the HAPAM and P(AM-*co*-DMC) confirm the presence of both hydrophobic and hydrophilic monomers in the copolymers (Figure 4). Previously, there were no reports regarding the thermal degradation of the P(AM-*co*-DMC) except for the PAM and some anionic copolymers. Therefore, in order to analyze the TG and DTG of the HAPAM and P(AM-*co*-DMC), we should consider the TGA of the PAM. M.J. Caulfield et al. [30] summarized a prevenient study, and they found the thermal degradation of the PAM divided into three regions. The first was the temperature up to 200 °C, where the PAM was generally thermally stable and underwent very little physical change, apart from a slight loss. Van Dyke and Kasperski [31] found that dried PAM had no weight loss before approximately 220 °C. The next region was between 200 °C and 300 °C, where it was found that the intra- and intermolecular [32,33,34] imidization reactions occurred via the pendant amide groups. The final region was above 300 °C, where the predominant reactions were the random bond scission of the polymeric main chain backbone that formed long-chain hydrocarbons. As shown in Figure 4a, when the temperature increased from 35 °C to 550 °C, a comparison among those data shows that the total weight loss of cw101 was the highest, reaching 75.87%. For the HAPAM, the general trend is that as the alkyl chain length increased, the total weight loss decreased. For example, the samples cw12-0.25, cw8-1, cw6-1 and cw4-1 have a total weight loss of 61.29%, 61.50%, 64.86% and 68.66%, respectively. Similarly, L.D. Cunha et al. [35] investigated the TG thermal curves of the surface modification of styrene-divinylbenzene copolymers by PAM grafting. They found that the total weight loss of the modified copolymer was lower. Feng et al. [20] showed that the thermal stabilities of the polymers increased due to the incorporation of a long-chain alkyl hydrophobic monomer.

**Figure 5:**
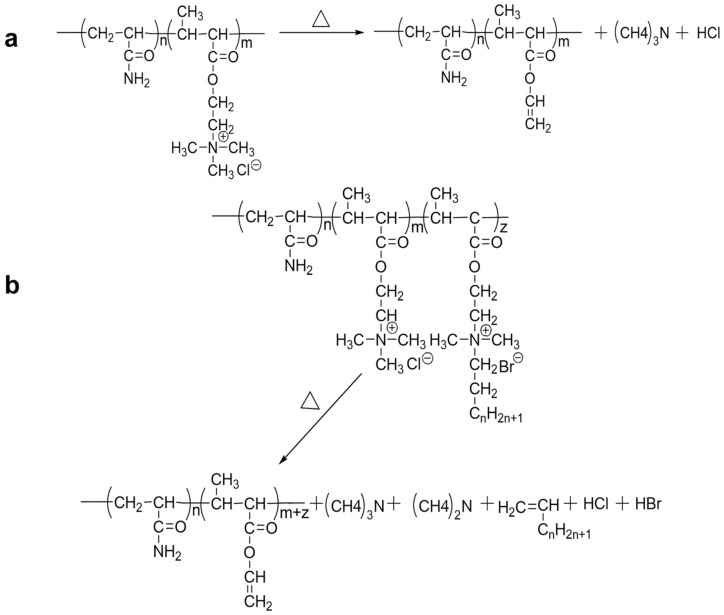
The Hofmann elimination reaction for (**a**) P(AM-*co*-DMC) and (**b**) HAPAM at high temperatures.

From the DTG thermal curves, it was observed that cw101 has three thermal degradation regions: 150–170 °C, 200–340 °C and 350–450 °C. The first thermal degradation region is between 150 °C and 170 °C, which is owing to the quaternary ammonium salt in the polymer chain that occurred due to a Hofmann elimination reaction (Figure 5a). In the second region, intra- and intermolecular [32,33,34] imidization reactions and the random bond scission of the polymeric main chain backbone occurred. In addition, ester pyrolysis reactions occurred in the final region.

By comparing the DTG thermal curves of cw101 and the HAPAM, it was found that the 200–250 °C peak in the HAPAM DTG thermal curves was the prominent difference with the cw101 curve (Figure 4b–f). The 150–170 °C peak moves toward a higher temperature when the alkyl chain of the hydrophobe increases in length; furthermore, this peak was not observed in cw6, cw8 and cw12. This difference indicates that the reaction depicted in Figure 5b in the HAPAM occurred at a higher temperature than the reaction depicted in Figure 5a.

### 2.3. Rheological Behavior

The influence of the hydrophobe on the solution behavior of the HAPAM has already been studied by some authors [8,9,10,12,13,14,15,36]. It has been shown that the hydrophobe can considerably enhance the solution viscosity and impart the solution-particular rheological properties, such as shear thinning, shear thickening and viscoelasticity. In this part, we investigated the influence of the hydrophobic monomers on the rheological solution behavior in terms of the copolymer microstructure and the type of hydrophobe used.

**Figure 6:**
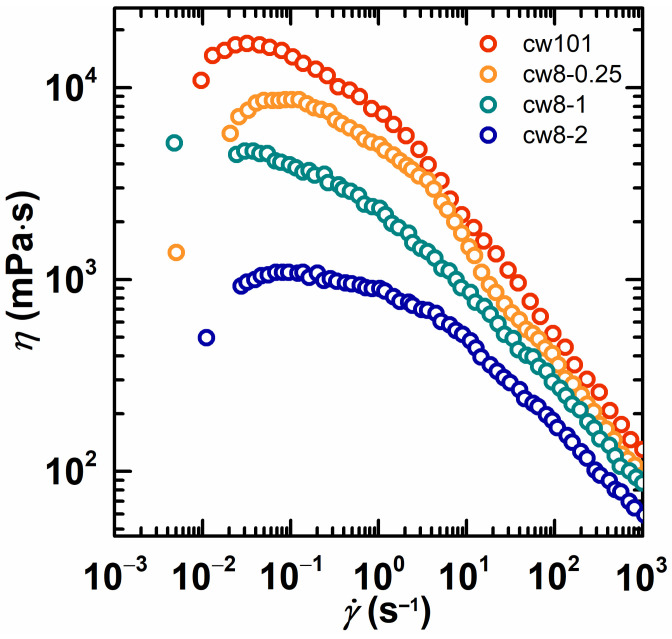
The apparent viscosity as a function of the shear rate for cw101 and cw8 with different hydrophobe concentrations. The copolymer concentration was fixed at 2 wt% in all solutions.

**Figure 7:**
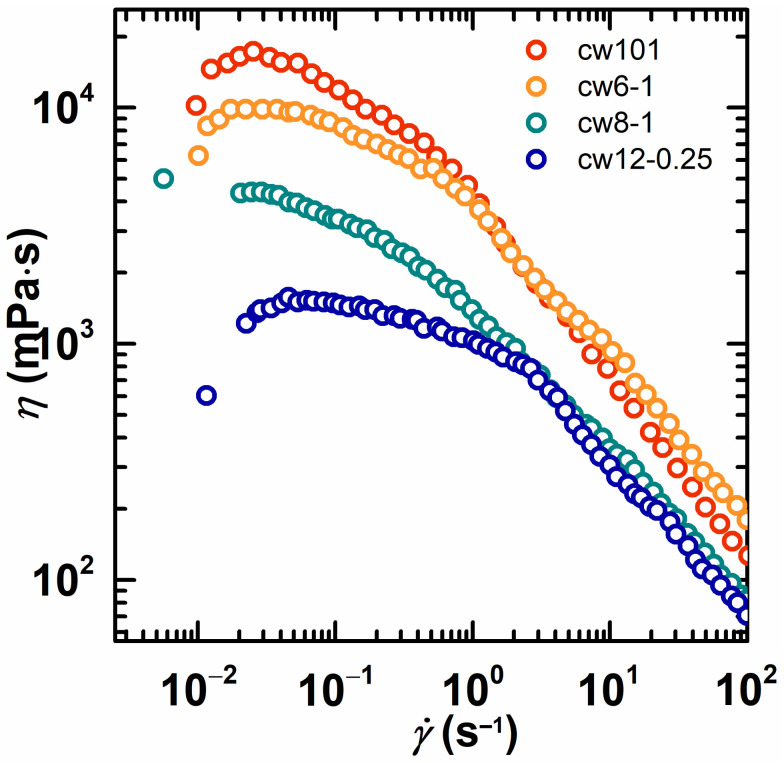
The apparent viscosity as a function of the shear rate for cw101 and HAPAM with different alkyl chains. The copolymer concentration was fixed at 2 wt% in all solutions.

Figure 6 and Figure 7 show the apparent viscosity as a function of the shear rate for the cw101 and HAPAM solutions. An expected shear thickening behavior of the HAPAM solutions was not observed. One possible reason is the higher copolymer concentration. This is supported by the findings of Vrahopoulou and McHugh [37], who predicted a disappearance of the shear thickening region with an increasing molecular weight and concentration, and it was confirmed experimentally by Jenkins et al. [38]. Furthermore, Volpert et al. [9] also did not observe the shear thickening behavior at higher polymer concentrations (2 wt%), except for the classical shear thinning behavior with an increasing shear rate. Another reason might be attributed to the formation of little blocks in the initial polymer chains (the hydrophobe content <2 mol%). Volpert et al. [9] believed that the large blocks formed in the initial polymer chains (hydrophobe content >>10%) were probably the result of a higher association.

For a copolymer, the viscosity changes behavior as the shear rate increases, and Figure 6 shows the influence of the content of the hydrophobe. The viscosity of cw101 (without hydrophobe) decreased quickly as the shear rate increased. In contrast, the viscosity decrease in cw8-0.25 (with 0.25 mol% hydrophobe) and cw8-1 (with 1 mol% hydrophobe) is slower, and cw8-2 has a significant flat region in the lower shear rate region. A conclusion can be drawn from Figure 6, which is that as the content of the hydrophobe increased, the shear thinning became weaker. Moreover, the shear thickening occurred when the content of the hydrophobe exceeded the critical content. This result reflects that copolymers have different microstructures. Similarly, Figure 7 shows the influence of the type of hydrophobe on the rheological properties. The HAPAM solution showed a notable difference as compared to the cationic PAM solution (cw101): the viscosity decrease in cw6-1, cw8-1 and cw12-0.25 was slower than that of cw101, as the shear rate increased. Therefore, the performance of cw12-0.25 is especially outstanding because the viscosity exhibited little change in the lower shear rate region. From Figure 7, a conclusion can be drawn that as the alkyl chain length increases, the shear thinning becomes weaker. In other words, the HAPAM with longer alkyl chains has stronger associations.

**Figure 8:**
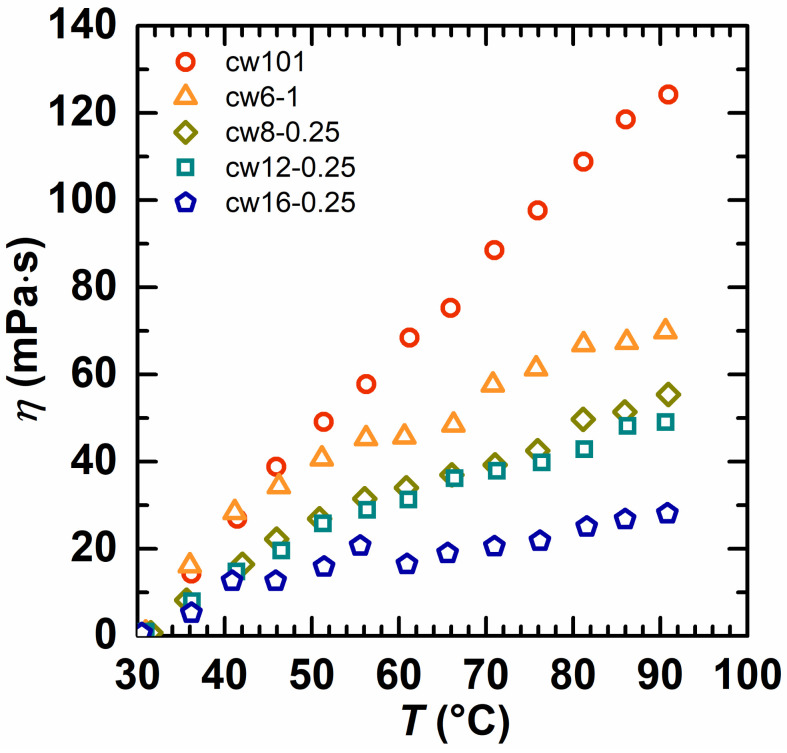
The reduced apparent viscosity as a function of temperature for cw101 and HAPAM with different hydrophobes (the copolymer concentration was fixed at 1 wt% in all solutions).

**Figure 9:**
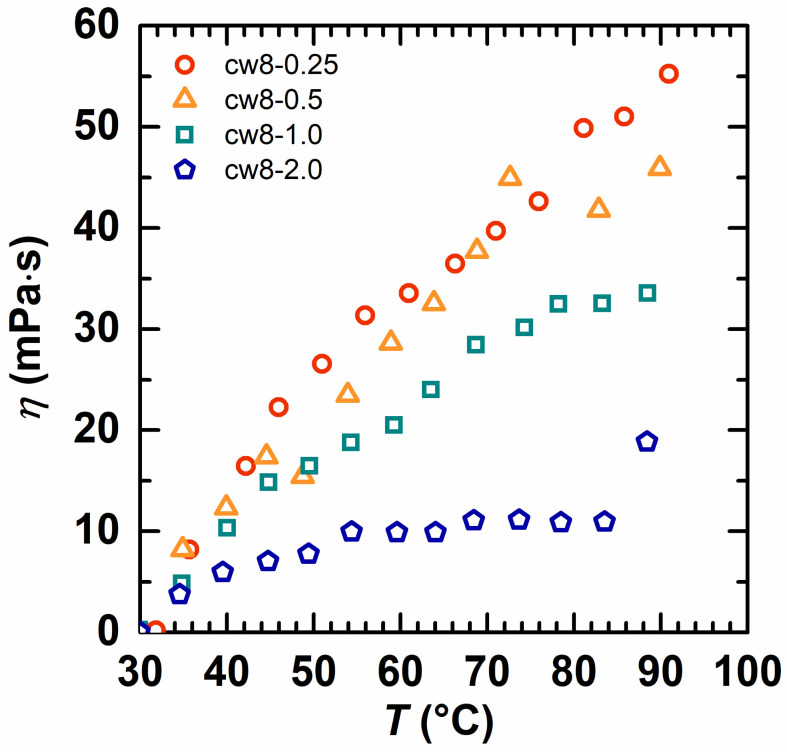
The reduced apparent viscosity as a function of temperature of HAPAM with different hydrophobic monomer concentrations. The copolymer concentration was fixed at 1 wt% in all solutions.

The effect of the temperature on the copolymer solution’s viscosity was investigated (Figure 8 and Figure 9). For a quick comparison, the reduced viscosity (defined as the viscosity difference between 30 °C and a higher temperature) as a function of temperature was obtained, and the polymer with a lower reduced viscosity held the strongest associativity [39]. From Figure 8, it is seen that the reduced viscosity of cw101 increased quickly with a temperature increase from 30 °C to 90 °C, but the viscosity reduction rate of the HAPAM was significantly lower. Moreover, the total reduced viscosity decreases with the hydrophobic alkyl chain length increase for the HAPAM. At a temperature of 90 °C, the total reduced viscosity was 124.42 mPa·s for cw101, 69.81 mPa·s for cw6-1, 55.38 mPa·s for cw8-0.25, 48.95 mPa·s for cw12-0.25 and 28 mPa·s for cw16-0.25. Thus, it is clear that as the hydrophobic alkyl chain length increases, the HAPAM association becomes stronger.

Figure 9 shows the reduced viscosity of the HAPAM with different hydrophobe concentrations when the temperature increased from 30 °C to 90 °C. One can find that the reduced viscosity decreased with the increase in the hydrophobe concentrations, and the polymer with a 2 mol% hydrophobe has the lowest value as 19.12 mPa·s, suggesting it has the best association.

Based on these experimental results, it was found that, in comparison with the cationic PAM (cw101), the HAPAM solutions exhibit unusual shear- and temperature-responsive rheological behavior. These phenomena suggest that there are significant differences in the polymer chain microstructure, and such differences are affected by both the molecular structure of hydrophobes and their content in copolymers. These findings are helpful to design and prepare HAPAM *w*/*w* emulsion products with the desired rheological properties, and these HAPAM *w*/*w* emulsion products can also reduce the polymer usage while keeping the similar rheological properties of the traditional PAM solution.

## 3. Experimental Section

### 3.1. Materials

Acrylamide (AM, 99.5%; Changjiu Agri-Scientific Co., Ltd., Nanchang, China), 2-methylacryloylxyethyl trimethyl ammonium chloride (DMC, 80%; Sanyo Chemical Ind. Ltd., Kyoto, Japan), dimethylaminoethyl methacrylate (DM, ≥99.5%; Shanghai WellTone Material technology Co., Ltd., Shanghai, China), alkyl bromine (≥98.0%; Chengdu Kelong Chemical Factory, Chengdu, China), acetone (AR; Guangdone Guanghua chemical Factory Co., Ltd., Guangzhou, China), (NH_4_)_2_SO_4_ (A. R. grade, Sinopharm Chemical Group, Shanghai, China), Na_2_SO_4_ (A. R. grade, Sinopharm Chemical Group, China), ammonium persulfate (APS, A. R. grade, Shanghai API China Chemicals Reagent Ltd., Shanghai, China), sodium bisulfite (A. R. grade, Shanghai API China Chemicals Reagent Ltd.) and 2, 2′-azobis(2-(2-inidazolin-2-yl)propane)-dihydro chloride (VA-044, Wako Pure Chemical Industries, Ltd., Osaka, Japan) were all used as received without further purification. The water was distilled three times to achieve a resistance of higher than 18 MΩ‧cm, and the purity of nitrogen was 99.999%.

### 3.2. Synthesis Hydrophobic Monomers

**Figure 10:**
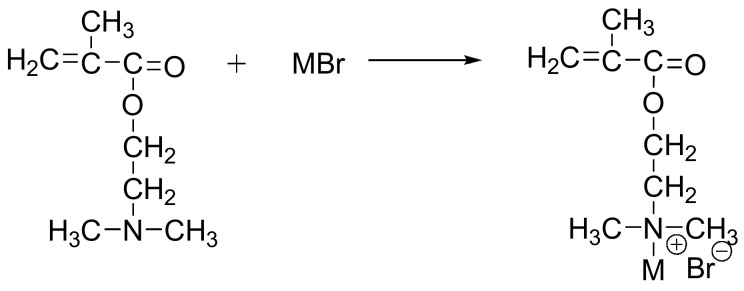
The synthetic route for preparation of hydrophobic monomer (M is *n*-alkyl).

Hydrophobic monomers were synthesized by quaternization between DM and *n*-alkyl bromine (Figure 10) [40]. The resulting hydrophobic monomers were named as DM-*n*. The number *n* in the hydrophobic monomers varied from 4 to 16 so that we could attain a wide range of samples.

**Figure 11:**
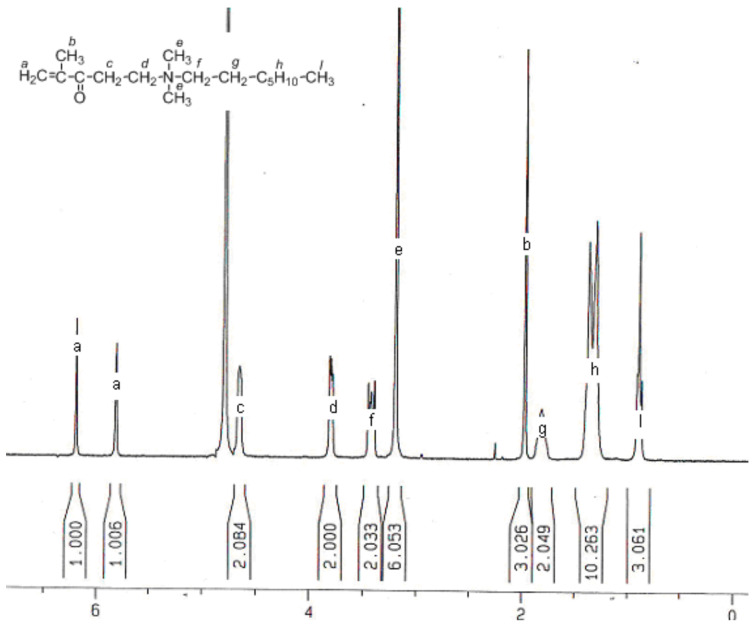
Typical ^1^H NMR spectrum of DM-8 hydrophobic monomers.

The process of synthesis and purification of hydrophobic monomers is as follows: 1.00 mol of DM and 1.05 mol of alkyl bromine were dissolved in 400–500 mL acetone, the reaction was sealed for one week at 25 °C. The system temperature was cooled to −12 °C to crystallize the hydrophobic monomers. The crystallized monomers were washed multiple times with cool acetone and then freeze-dried under vacuum to afford the product. The monomer molecular structure was characterized with NMR spectroscopy. A typical ^1^H NMR spectrum of DM-8 is shown in Figure 11. One can find that all peaks were attributed to corresponding protons, indicating the correct molecular structure.

### 3.3. Dispersion Polymerization of HAPAM

**Figure 12:**
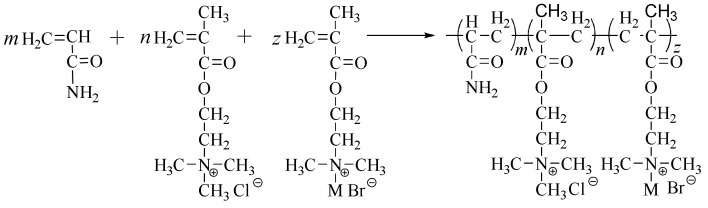
The synthetic route for preparation of HAPAM.

The copolymers of AM, DMC and hydrophobic monomers were synthesized by aqueous dispersion polymerization. The synthetic route is shown in Figure 12. A designated amount of monomers (AM, DMC and hydrophobic monomers), inorganic salt, stabilizer and deionized water were combined in a 500 mL, four-necked round-bottom flask equipped with a water condenser, thermometer, mechanical stirrer and N_2_ inlet and outlet. The dispersion polymerization was conducted at 25 °C in N_2_ atmosphere with a stirring speed of 800–1000 rpm. After purging with N_2_ for 15 min, the solutions of (NH_4_)_2_S_2_O_8_, NaHSO_3_ and VA044 were injected. The polymerization was initiated after 20–30 min. For all the dispersion polymerizations, the total concentration of the monomers was fixed at 10 wt%, while the content of hydrophobic monomers in the polymer chains varied from 0.25 mol% to 2 mol%. The total concentration of inorganic salt was fixed at 32 wt%. Polymerization proceeded for 16 h, resulting in milk-like dispersions that were called “*w*/*w*” hydrophobically associating polyacrylamide emulsions.

### 3.4. Ion Change Chromatography (IC), Thermogravimetric Analysis (TG) and Differential Thermal Gravity (DTG)

The polymers were separated from the emulsions and then washed successively with acetone in order to remove residual monomers. The purified polymers were dried under vacuum at 45 °C, and designated amounts of dried polymers were taken and burnt in an oxygen bottle. The giblets were absorbed with a mixture of NaOH and H_2_O_2_ and diluted to a designated volume with deionized water. An Ion Chromatography System (ICS-90, Dionex, Sunnyvale, CA, USA) was utilized to determine the bromine content in the copolymers. The thermogravimetric analysis was carried out on an SDT Q600 machine (NICOLET, Madison, WI, USA) under nitrogen flow. Samples with 5 mg weight were heated from 35 to 550 °C with a constant heating rate of 20 °C/min. The DTG data were obtained simultaneously, which was used to determine the enthalpy change in polymers as a function of temperature.

### 3.5. Measurement of Intrinsic Viscosity

Concentrated stock polymer solutions were prepared by dissolving appropriate amounts of the purified polymers in a mixed aqueous solution of 0.5 M NaCl and 0.5 M formamide with gentle magnetic agitation. The intrinsic viscosity [*η*] of all the polymers was measured at 30 ± 0.2 °C which was maintained with a thermostatic water bath. The stock solutions were successively diluted with the same mixed aqueous solutions by the five-spot method. The measurements were carried out using an Ubbelhode capillary glass viscometer with a capillary inner diameter of 0.56 mm.

### 3.6. Observation of the Particles in Polymer Dispersion

The morphology and size of the polymer dispersion particles were observed by an optical microscope (Axioskop 40, Zeiss, Jena, Germany).

### 3.7. Determination of HAPAM Solubility in Water

The solubility was evaluated by the time where the apparent viscosity reached a stable value. The apparent viscosity of the polymer solutions was measured by a Brookfield LVDV-III Programmable rheometer equipped with the 31^#^ spindle and corresponding adaptor at 20 °C. A 10 mL *w*/*w* emulsion was dissolved in 90 mL deionized water with a 300 rad/min magnetic agitation. The apparent viscosity of the polymer solutions was measured every 20 s until it reached a stable value.

### 3.8. Rheological Measurements

The emulsions were dissolved in deionized water and gently stirred for 1–2 h. For the viscosity–temperature curves, the data were obtained with a Brookfield LVDV-III programmable rheometer equipped with the 31^#^ spindle. For the viscosity–shear rate curves, the data were obtained with a Physical MCR300 (Anton Paar, Rannachstrasse, Austria) controlled stress rheometer equipped with a concentric cylinder geometry (CC27 cup radius 14.46 mm) at 25 °C.

## 4. Conclusions

In summary, this paper reports the preparation of HAPAM “water-in-water” emulsions using an aqueous dispersion polymerization technique. All these emulsions can be stored more than 6 months, showing excellent stability. IC, TG and DTG techniques were used for a qualitative analysis of the polymer composition, and the presence of hydrophobic monomers in the copolymers was shown. The polymer particles in the emulsions were well dispersed, and their size was around 5 μm. All these HAPMA emulsions can be completely dissolved in water in 180 s forming a homogeneous clear solution, showing the advantage of rapid dissolution. Moreover, the influence of the hydrophobe on the solution behavior of the HAPAM was examined. It was found that the solution behavior of the HAPAM is different from the corresponding polymer without a hydrophobic monomer. These HAPAM solutions show a better shear and temperature resistance due to the association behavior, which is affected by the molecular structure of hydrophobes and their content in copolymers. Based on the excellent stability, solubility and rheological properties, we believe that these HAPAM *w*/*w* emulsions could find widespread applications. Future researchers in this area may focus on simplifying the synthesis procedure and further improving the solubility of the emulsion.

## Figures and Tables

**Table 1:** The recipes and characters of the copolymers.

Simple Code ^a^	Monomers				Intrinsic Viscosity [η] (mL/g)
	AM (wt%)	DMC (wt%)	Hydrophobic Monomer		
			Code	Content (mol%) ^b^	
CW4-1	5.00	5.00	DM-4	1.00	757
CW6-1	5.00	5.00	DM-6	1.00	590
CW8-0.25	5.00	5.00	DM-8	0.25	1020
CW8-0.5	5.00	5.00	DM-8	0.50	871
CW8-1	5.00	5.00	DM-8	1.00	702
CW8-2	5.00	5.00	DM-8	2.00	531
CW12-0.25	5.00	5.00	DM-12	0.25	638
CW16-0.25	5.00	5.00	DM-16	0.25	542
CW101	10.00	10.00	—	—	1231
CW206	10.00	10.00	—	—	—

^a^ The sample code refers to the content and alkyl group of the hydrophobic monomer. ^b^ Hydrophobic content in the polymer.

## Data Availability

The data presented in this study are available on request from the corresponding author.

## Sample Availability

Samples of the compounds are available from the authors.
